# Closantel; a veterinary drug with potential severe morbidity in humans

**DOI:** 10.1186/s12886-016-0387-x

**Published:** 2016-11-29

**Authors:** Seyed Ali Tabatabaei, Mohammad Soleimani, Mohammad Reza Mansouri, Ahmad Mirshahi, Bahman Inanlou, Mojtaba Abrishami, Ahmad Reza Pakrah, Hamideh Masarat

**Affiliations:** Eye Research Center, Farabi Eye Hospital, Tehran University of Medical Sciences, Tehran, Iran

**Keywords:** Closantel, Side effect, Visual loss, Case

## Abstract

**Background:**

Closantel is a halogenated salicylanilide with a potent anti parasitic activity. It is widely used in management of parasitic infestation in animals, but is contraindicated in humans.

**Case presentation:**

A 34-year-old man with depression was referred to our center with progressive loss of vision in both eyes 10 days after unintentional ingestion of three 500 mg tablets of Closantel. On fundus examination, left optic disc margin was blurred. His bilateral visual acuity was no light perception (NLP) despite prescribed IV erythropoietin injections 20,000 units daily for 3 days and 1gr intravenous methylprednisolone acetate for 3 days followed by 1 mg/kg oral prednisolone. On macular optical coherence tomography (OCT), a disruption in outer retina was observed. Electroretinogram and visual evoked potential tests showed visual pathway involvement.

**Conclusions:**

Destruction of neurosensory retina and visual pathways after accidental Closantel use is related to severe visual loss. This case alerts us about the destructive effect of this drug on humans even in low dosage which necessitates preventive efforts to reduce the chance of this morbid side effect.

## Background

Closantel is a halogenated salicylanilide with a potent anti parasitic activity, which probably acts mainly via energy metabolism pathway by uncoupling oxidative phosphorylation in liver flukes. It is widely used in management of parasitic infestation in animals but is contraindicated in humans and in high dose in milk producing animals because of its secretion in milk. In this report we present a 34-year-old man who was referred to tertiary referral ophthalmology hospital, with the chief complain of progressive loss of vision in both eyes after taking Closantel. What is interesting in this case is severity of visual loss after taking this veterinary drug. Another unique point is blindness following only one bolus dose of 1500 mg Closantel which has not been reported in previous similar reports.

## Case presentation

**Fig. 1:**
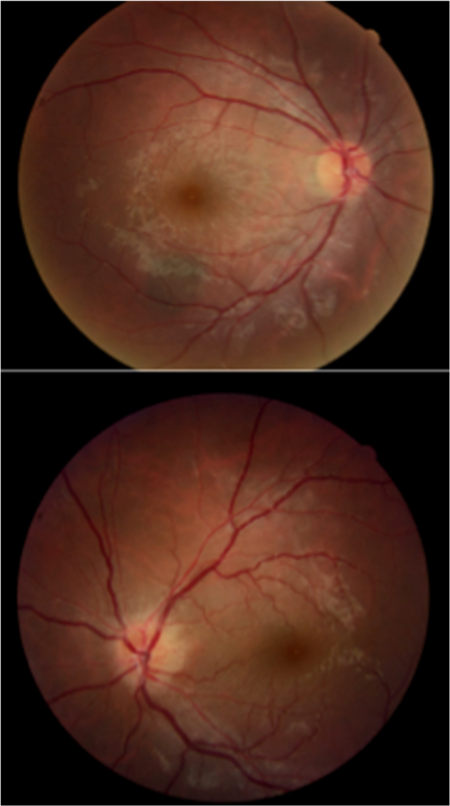
Fundus photo at presentation; 10 days after ingestion

**Fig. 2:**
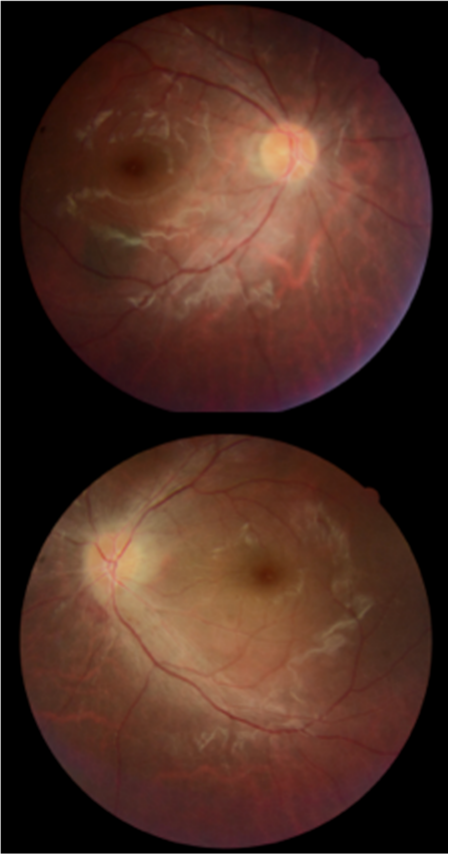
Fundus photo show the start of optic disc pallor (26th day)

A 34-year-old man with previously diagnosed depression was referred to our emergency department with progressive loss of vision in both eyes and mild headache following unintentional ingestion of three 500 mg tablets of Closantel 10 days before admission. Written informed consent was obtained from the patient for publication of this case report and accompanying images. A copy of the written consent is available for review. The patient mentioned that his vision (that was previously normal) started to deteriorate 3 days after ingestion. His past medical history was negative for any other ophthalmic or systemic disorders and he and his family did not mention taking drugs or exposure to other toxins. Systemic examination and brain imaging (high-resolution contrast-enhanced MRI imaging of his retrobulbar visual pathways and cortex) did not show any positive findings. On fundus examination, optic disc swelling was present in the left eye (Fig. 1). His visual acuity was light perception and no light perception (NLP) in right and left eyes, respectively. His visual acuity showed no response to IV injections of erythropoietin 20,000 units daily for 3 days and 1gr intravenous methylprednisolone acetate for 3 days followed by 1 mg/kg oral prednisolone for 2 weeks. On discharge day, both visual acuities were NLP. Figure 2 shows his fundus photos 26 days after ingestion; unfortunately, he did not continue his visits after that time because of no improvement in his vision.

**Fig. 3:**
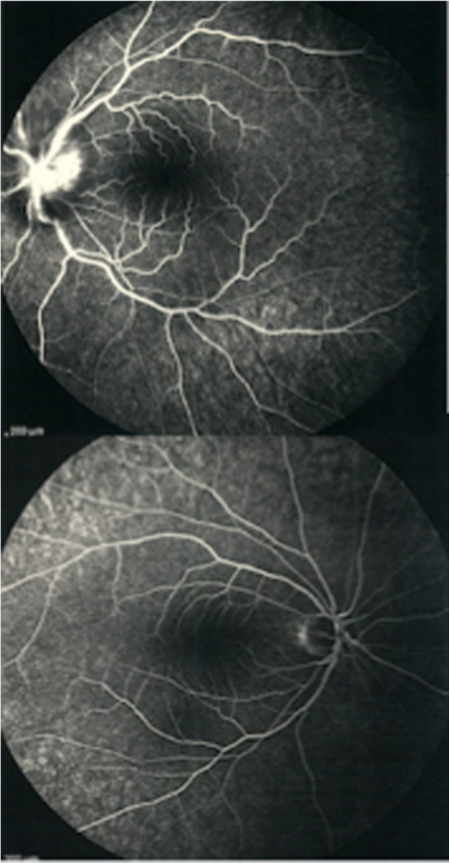
Fluorescein angiography of both eyes, showing disc leakage in the left eye

Fundus fluorescein angiography disclosed mild optic nerve head hypoflourescenc in early phases due to edema and leakage of dye during late phases in left optic disc (Fig. 3).

**Fig. 4:**
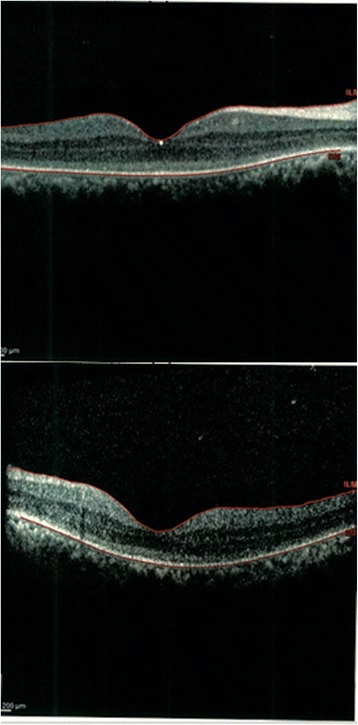
OCT of both eyes, showing outer retinal layer destruction

**Fig. 5:**
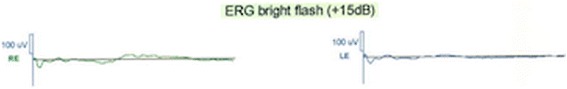
ERG show severe decrease in retinal responses

**Fig. 6:**
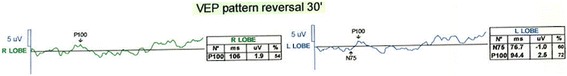
There is a significant decrease in amplitude and latency of VEP in both eyes

On macular optical coherence tomography (OCT), a disruption in outer retina was seen in both eyes (Fig. 4). Electroretinogram (ERG) showed severe decrease in both rod and cone responses (Fig. 5) (according to the manufacturer’s guidelines: http://www.metrovision.fr). In visual evoked potential (VEP) test, there was a significant decrease in amplitude and latency of VEP in both eyes (Fig. 6) (according to the manufacturer’s guidelines: http://www.metrovision.fr). Comprehensive infective and inflammatory tests (including Aquaporin 4 serology and cerebrospinal fluid analysis by lumbar puncture) did not show any positive finding. All other laboratory tests were within normal range except for a mild degree of normocytic normochromic anemia (serum hemoglobin, 11 mg/dl) and increase in liver aminotransferases (alanine aminotransferase (ALT) and aspartate aminotransferase (AST)) more than two times.

Closantel is a widely used veterinary anti helminthic drug which is active against Fasciola and Haemonchus species [1]. Toxicity against central nervous system, optic nerve and retina is a well-known adverse effect in sheep and gouts [2–4]. However, to the best of our knowledge only four reports have been previously published in English literature addressing Closantel toxicity in humans [5–8]. An outbreak of Closantel toxicity was first described in Lithuania in 90s [5]. Two sporadic similar cases have also been reported in Morocco. [6, 7] Inadvertent use of Closantel was the cause of toxicity that led to blindness in these reports. Partial recovery of visual acuity after plasma exchange in a man, who ingested Closantel because of parasite fearing, has been reported recently [8]. However there are other previous reports showing unconfirmed role of plasmapheresis for treatment of Closantel toxicity causing blindness [1].

Spongiosis of brain white matter and spinal cord is the most significant finding in microscopic pathology in case of Closantel toxicity in animals [2]. These changes are symmetrical and affect periventricular white matter, optic radiation, brain stem, thalamic nuclei and cerebellar peduncles. The effect of Closantel on the central nervous system, optic nerve and retina is a toxic and irreversible effect. The induced histopathological changes of optic nerve caused by Closantel toxicity is represented by a significant spongiform change, edema and vacuolization of the myelin resulting in optic disc atrophy. In retina, it leads to necrosis and apoptosis of the outer retinal layers especially photoreceptive cells [6]. In addition, diffuse optic nerve vacuolation may occur that mainly develops in the intracranial portion of optic nerve rather than intraorbital part. Pathological study of the retina reveals severe necrosis in photoreceptor layer, outer and inner nuclear layers and outer plexiform layer, which explains the direct toxic effect of Closantel on the retinal tissues [3]. The term status spongiosis in animals refers to spongy vacuolation of white matter seen by light microscopy. Electron microscopy demonstrates intracellular, extracellular and intra myelinic accumulation of fluid [2]. The exact pathogenesis of myelin spongiosis is not well understood.

Myelin splitting as a result of some exogenous or endogenous substances intoxication such as nitrobenzene is a well-described condition in sheep and goats [2, 9, 10]. Nitrobenzene which acts by uncoupling of mitochondrial Phosphorylation results in similar changes in oligodendrocytes [11]. Closantel is a salicylanilide drug which mainly interrupts oxidative Phosphorylation in helminthes [1]. Due to the similarity in mechanism of action, myelin spongiosis might be a possible explanation for Closantel toxicity. Destruction of neurosensory retina and visual pathways which was obvious in OCT, ERG and VEP is compatible with pathologic findings in animal reports [3, 12]. Other signs of central nervous system involvement such as ataxia and paresis have been described in animals [3, 12]. Although partial recovery of visual acuity after plasma exchange in a man, who ingested Closantel because of helminthiasis fearing, has been reported recently, it seems that this may be related to the natural course of toxicity; because the effect is toxic and probably irreversible [8].

## Conclusions

Severe reduction in visual acuity was the main presenting picture in our patient. We didn’t observe any accompanied central nervous system symptoms. Brain imaging was normal. Mild anemia and liver transaminase enzymes elevation were the only systemic complications. However, such involvements have not been mentioned in human reports, perhaps due to lower amount of ingestion. Unfortunately, the patient did not come to us after the 26th day post treatment because of not improving in his vision, thus we could not record any other imaging after 1 month. However, we could infer the destruction in outer retina according to OCT and possibly visual pathways according to VEP and ERG; all of them could confirm toxic optic neuropathy. Interestingly, although right optic disc did not show significant swelling but OCT, ERG and VEP were compatible with decreased vision in the right eye. A shocking point in our case is blindness following only as low as one bolus dose of 1500 mg Closantel that has not been reported in previous reports. Treatment with high doses of intravenous methylprednisolone acetate and IV erythropoietin failed to improve visual outcome in our patient. Direct toxic effect of the drug on retinal and neural tissues, not the host inflammatory response, might be an explanation for treatment failure. This case alerts us about the destructive effect of this drug on humans even in such low dosage which necessitates preventive efforts for this morbid side effect.

## Abbreviations

ALT: Alanine aminotransferase
AST: Aspartate aminotransferase
ERG: Electroretinography
OCT: Optical coherence tomography
VEP: Visual evoked potential
